# Cross-cultural adaptation and psychometric properties of the Vietnamese version of the Lower Extremity Functional Scale (LEFS) in individuals with knee osteoarthritis

**DOI:** 10.1186/s41687-026-01088-z

**Published:** 2026-05-19

**Authors:** Van Phat Huynh, Thi Diep Tran, Komsak Sinsurin, Chutima Jalayondeja

**Affiliations:** 1 Ergonomic and Physical Activity Research Unit, Faculty of Physical Therapy, Mahidol University, Nakhon Pathom, Thailand; 2 Faculty of Rehabilitation, Hong Bang International University, Ho Chi Minh City, Vietnam; 3 Biomechanics and Sports Research Unit, Faculty of Physical Therapy, Mahidol University, Nakhon Pathom, Thailand

**Keywords:** Lower Extremity Functional Scale (LEFS), Knee osteoarthritis, Cross-cultural adaptation, Psychometric properties

## Abstract

**Objective:**

To translate, cross-culturally adapt, and examine the psychometric properties of the Vietnamese version of the Lower Extremity Functional Scale (LEFS-V) in individuals with knee osteoarthritis (KOA).

**Design:**

A cross-sectional study.

**Setting:**

Two hospitals and two Catholic convents in Vietnam.

**Participants:**

174 Vietnamese individuals aged ≥ 45 years with KOA were diagnosed based on clinical examination using the National Institute for Health and Care Excellence (NICE) criteria.

**Main outcome measures:**

Participants completed the baseline assessment and 5-7days retest, which included the LEFS-V, Numerical Pain Rating Scale (NPRS), and Knee Injury and Osteoarthritis Outcome Score (KOOS). Psychometric property testing included content validity (CVI), internal consistency (Cronbach’s α), test–retest reliability (ICC _(3,1)_, standard error of measurement (SEM), minimum detectable change (MDC95), and construct validity. Spearman correlations were used to examine associations with the KOOS subscales and NPRS.

**Results:**

Strong content validity with item-CVI from 0.83 to 1.00. LEFS-V demonstrated high internal consistency (Cronbach’s α = 0.942) and excellent test–retest reliability (ICC _(3,1)_ = 0.985; 95% CI: 0.974–0.991), SEM = 1.4 and MDC_95_ = 3.88. LEFS-V correlated positively with KOOS-Pain (r_s_ = 0.59), KOOS-Symptoms (r_s_ = 0.30), KOOS-ADL (r_s_ = 0.71), KOOS-Sport/Rec (r_s_ = 0.50), and KOOS-QoL (r_s_ = 0.18), and negatively with NPRS (r_s_ = -0.63).

**Conclusion:**

The LEFS-V is a reliable and valid scale for assessing lower extremity functional status in Vietnamese individuals with KOA.

## Background

Musculoskeletal disorders (MSDs) are the leading cause of disability worldwide, with an estimated 1.71 billion people affected and 528 million people suffering from osteoarthritis according to the Global Burden of Diseases (GBD) 2019 data [1, 2]. Knee osteoarthritis (KOA) is the most common type of osteoarthritis, and its severity significantly affects mobility and quality of life, particularly among older adults. The global prevalence of KOA was estimated at 16% among individuals aged ≥ 15 years and nearly 23% among those aged ≥ 40 years [3]. In Vietnam, the prevalence of this condition is also rising due to population aging and lifestyle changes associated with increasing rates of overweight and obesity [4]. The study by Ho Pham et al. reported that the prevalence of KOA increased with advancing age, doubling from 30% in individuals aged 40–49 years to 61% in those aged ≥ 60 years [4]. The burden not only affects individuals’ lives but also places great pressure on healthcare systems and society as a whole. Therefore, these impacts emphasize the importance of enhancing accurate assessment of lower limb function in patients with KOA.

A feasible and standardized outcome measure that captures patients’ perceptions of their symptoms and functional status related to daily activities is essential for individuals with KOA. A patient-reported outcome measure (PROM) is one type of instrument designed to capture patients’ perspectives and to inform clinical decision-making for management, particularly in physical therapy. The Lower Extremity Functional Scale (LEFS), developed by Binkley et al. (1999), focuses on lower limb functions during activities of daily living, including sitting, standing, walking, and running [5]. LEFS is widely recognized for its reliability and validity in assessing lower limb function in KOA and other musculoskeletal conditions [5]. Although the Vietnamese version of KOOS is also widely used to evaluate patients with knee osteoarthritis, it mainly assesses more general aspects rather than focusing on overall function of the lower limb. Moreover, the KOOS includes multiple subscales, which may increase respondent burden and reduce feasibility for routine clinical practice. In contrast, the LEFS is a concise, unidimensional measure focusing specifically on lower-limb physical performance across various daily and physical activities, making it more suitable for evaluating overall functional recovery in patients with KOA. Similar to the original KOOS, KOOS-12 contains fewer items, it was developed to capture multiple domains of knee-related health status, including symptoms, pain, activities of daily living, sport and recreation function, and knee-related quality of life [6]. Therefore, KOOS-12 reflects knee-specific health status across several domains, whereas the LEFS more directly focuses on functional activity limitations of the lower limb. For this reason, the LEFS was considered more suitable for our expectation. To ensure linguistic and cultural appropriateness and also reduce the respondent bias, the LEFS has been translated and adapted into multiple languages, including Italian, Dutch, Spanish, Brazilian Portuguese, German, Serbian, Chinese, and Arabic, thereby facilitating its global application [7–13].

All versions have demonstrated very high reliability and validity. Cronbach’s α coefficients indicated excellent internal consistency (> 0.90) and intraclass correlation coefficients (ICC) for test-retest reliability are also very high (> 0.90). Factor analysis showed that the LEFS primarily measures a unidimensional factor. For example, in the Spanish version [10], one factor accounted for 85% of the total variance, although some versions reported the presence of a second factor, such as the German and Persian versions [13, 14]. In terms of convergent validity, LEFS scores correlated strongly with relevant physical function and pain scales, with correlation coefficients ranging from 0.5 to 0.9 for instruments such as the SF-36 and WOMAC. A similar pattern was observed across all versions, where the LEFS showed low correlations with unrelated constructs such as mental health (psychological component of the SF-36; *r* = 0.2–0.4), thereby demonstrating good discriminant validity. These findings across LEFS translations highlighted the potential influence of cultural and linguistic differences on item comprehension and response accuracy, while preserving conceptual equivalence with the original instrument.

In Vietnam, there is currently no official Vietnamese version of the LEFS that has been translated, culturally adapted, and tested for psychometric properties such as reliability, validity, and responsiveness. This gap needs to be addressed to prevent dependence on non-standardized tools for assessing lower limb function, which may increase the risk of bias, hinder data comparability, and limit the generalizability of research findings. Therefore, this study aimed to translate the LEFS into Vietnamese and evaluate the psychometric properties of the translated version. This study examined the content validity, as well as the test-retest and internal consistency reliability of the LEFS-Vietnamese version in individuals with KOA. We expected that our results would provide a solid scientific foundation for the application of LEFS-Vietnamese in clinical and research settings, thereby contributing to more effective management of KOA and other lower limb musculoskeletal disorders.

## Method

This study was conducted over a 9-month period, from September 2024 to May 2025. Data were collected from individuals with KOA who were registered at two hospitals and two Catholic convents: the Ho Chi Minh City Traditional Medicine Hospital, the Le Van Thinh Hospital, the Congregation of Our Lady of the Visitation of Hue, and the Congregation of the Lovers of the Holy Cross. Permission was obtained from the original developer of the LEFS, Dr. J. M. Binkley, to perform its cross-cultural adaptation. Ethical approval for this study was granted by the Medical Ethics Committee of Hong Bang International University (Approval No. 04/PCT-HĐĐĐ-ĐH).

### Phase I: Translation and cross-cultural adaptation of the LEFS

**Fig. 1:**
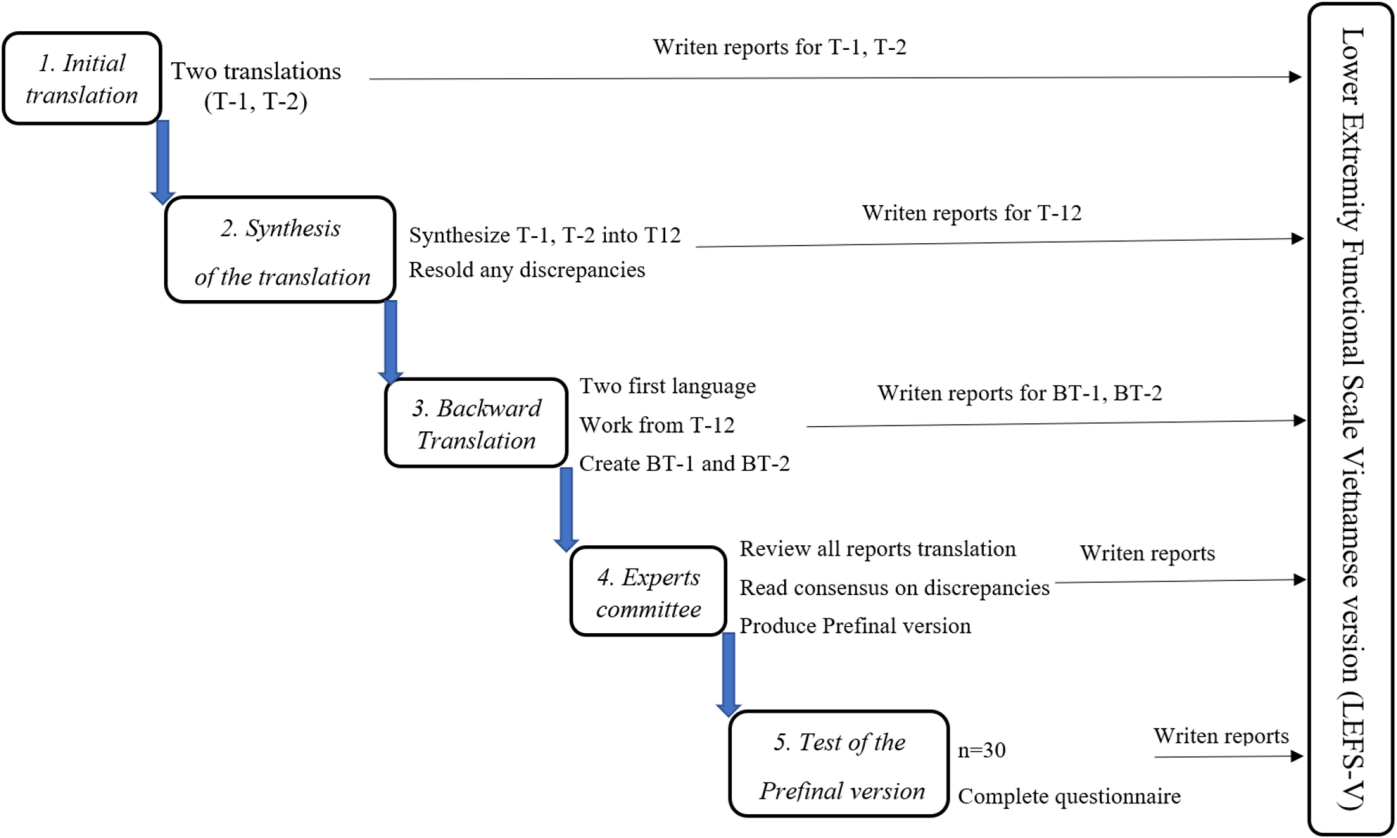
Translation and cross-cultural adaptation of LEFS

The five stages of translation and cross-cultural adaptation were conducted based on Beaton’s health-related translation guidelines [15], as shown in Fig. 1. In step1, the forward translation was performed by two native Vietnamese translators (T1 and T2). They were a PhD candidate (T1) and a master of physiotherapy (T2), both fluent in English and Vietnamese. In step 2, a consensus version was developed after a meeting between the research team and the two translators to resolve any discrepancies. In step 3, backward translation was conducted by two native English translators who had lived in Vietnam for 12 and 15 years, respectively (BT1: mechanical engineer; BT2: pharmacist). They independently translated the synthesized Vietnamese version back into English. Neither translator had prior exposure to or experience with the original LEFS questionnaire. In Step 4, the committee involved in the translation and cultural adaptation process consisted of eight experts with linguistic and clinical expertise. Specifically, an English language specialist and a physiotherapist performed the forward translation (TI and T2); a mechanical engineer and a pharmacist performed the backward translation (BT1 and BT2); three physiotherapists, two of whom had over 15 years of clinical experience in musculoskeletal field at the hospital, including the treatment of KOA; a lecturer with musculoskeletal instructor in university; and a Vietnamese language specialist. The committee ensured semantic, idiomatic, experiential, and conceptual equivalence between the original and Vietnamese versions, producing the pre-final version of the LEFS. In Step 5, the pre-final version was pilot tested with Physiotherapist experts (*n* = 12) and individuals with KOA (*n* = 10) who met the study criteria. The pre-final LEFS was examined for content validity regarding clarity, relevance, and cultural appropriateness of each item. Based on their feedback, the final Vietnamese version (LEFS-V) was finalized.

### Phase II: Psychometric property testing

#### Participants

Individuals with KOA who were aged over 45 years were screened for eligibility. All participants were confirmed to have KOA through clinical examination using the National Institute for Health and Clinical Excellence (NICE) criteria [16]. Participants who volunteered and were diagnosed with KOA based on medical records participated in this study. Individuals were excluded if they had neuromuscular disorders, other musculoskeletal conditions, partial or total knee replacements, or cognitive impairments. All participants were informed about the study protocol and provided written informed consent prior to data collection. According to the COSMIN guidelines [17], a total of 174 participants were included.

#### Outcome measures

The Lower Extremity Functional Scale (LEFS) was developed by Binkley et al. (1999) to assess lower limb function in patients with musculoskeletal disorders. The scale consists of 20 items, each scored on a 5-point Likert scale ranging from 0 (extremely difficult or impossible) to 4 (no difficulty). The total score ranges from 0 to 80, with higher scores reflecting better function. Participants are asked to recall and select responses based on their ability to perform activities over the past 7 days. The original version of the LEFS has demonstrated high reliability, validity, and responsiveness in various clinical settings and has been translated into multiple languages [5, 7, 8, 10–14, 18]. This study translated the LEFS into Vietnamese (LEFS-V) and evaluated its psychometric properties to provide a standardized instrument for assessing lower-limb functional limitations in Vietnamese-speaking patients, which can be used in both clinical practice and research settings.

The Numeric Pain Rating Scale (NPRS) is a self-reported measure of pain intensity widely used in both clinical and research settings. The scale consists of 11 points (0–10), where higher scores indicate greater pain intensity. Pain intensity can also be categorized into subgroups: 1–3 represents mild pain, 4–6 indicates moderate pain, and 7–10 represents severe pain [19–21].

The Knee Injury and Osteoarthritis Outcome Score (KOOS) is a self-administered questionnaire developed by Roos et al. (1998) to assess knee-related problems in patients with knee trauma and osteoarthritis. It consists of 42 items across five subscales: KOOS-Pain (9 items), KOOS-Symptoms (7 items), KOOS-ADL (17 items), KOOS-Sport/Rec (5 items), and KOOS-QoL (4 items). Each item has a five-point Likert scale with response options ranging from 0 (no difficulty) to 4 (extremely difficult). Each subscale score is calculated using the formula: KOOS = 100 – [(mean of subscale items scores)/4 × 100], with higher scores indicating better knee function and fewer symptoms. The KOOS subscale scores range from 0 (worst) to 100 (best) [22]. The KOOS has been translated into Vietnamese, as KOOS-V. The KOOS-V was culturally adapted and validated for the Vietnamese population by Le et al. [23], demonstrating satisfactory validity and reliability for assessing patients with KOA in Vietnam [23].

#### Procedure

In each setting, two research assistants were assigned to collect data through face-to-face interviews. They were physiotherapists with 5 to 15 years of clinical experience and 4 to 10 years of experience working with patients with osteoarthritis. Data collection included demographic information and KOA-related assessments, including the LEFS-V, KOOS-V, and pain intensity using the NPRS. Demographic data including age, gender, weight, height, education level, occupation, comorbidities, affected side, and duration of KOA-were collected by two final-year physiotherapy students. All assessors received adequate training and practice in administering all questionnaires prior to data collection.

In the initial assessment, participants were asked to complete all questionnaires including LEFS-V, KOOS-V and NPRS. Participants completed the questionnaire through a face-to-face interview with a highly trained physical therapist to ensure accurate and thorough understanding of the questions. Data from the initial assessment were used to assess internal consistency and construct validity. The reassessment was conducted 5–7 days after the initial assessment The 5–7 day interval was selected based on previous studies, which recommend this timeframe to balance two key considerations: minimizing recall bias while ensuring that the clinical status of participants remains relatively stable between the two assessments [15, 17, 24]. Several previous studies that translated the LEFS have used similar retest intervals, including the Italian version (2 days) [8], the Spanish version (2–5 days) [10], and the Brazilian version (2 days) [12]. During the 5–7 days period, participants were instructed to continue their usual daily activities and received routine rehabilitation care at the hospital’s Faculty of Rehabilitation. No specific restrictions were imposed during this period. All participants were reminded of the date and time of reassessment (LEFS-V and NPRS) via telephone. Based on previous studies that recommended an optimal time interval between assessments, participants whose NPRS scores changed by 2 points or more at reassessment were excluded from the test–retest reliability analysis [23, 25, 26].

#### Data analysis

Data were analyzed using IBM SPSS Statistics version 30 (IBM Corp., Armonk, NY, USA) and were described as mean ± standard deviation (SD) for continuous variables and as frequency and percentage (%) for categorical variables. Demographic variables included age (years), gender (male/female), body mass index (BMI; kg/m²), education level (primary, secondary, high school, or university and above), occupation (manual work, office work, or retired), comorbidity (yes/no), affected side (left, right, or both), and duration of KOA (years).

For the content validity assessment, twelve experts working in the field of rehabilitation were invited to evaluate the clarity, relevance, and cultural adaptability of each item in the pre-final version of the LEFS. To quantify expert agreement, the Content Validity Index (CVI) was employed. Each expert rated all 20 translated items in the final LEFS-V based on two criteria: relevance and clarity. Relevance was assessed using a 5-point Likert scale ranging from 1 (“Highly irrelevant”) to 5 (“Highly relevant”), and clarity was assessed using a 5-point Likert scale ranging from 1 (“Completely unclear”) to 5 (“Very clear”). The Item-level Content Validity Index (I-CVI) was calculated as the proportion of experts rating each item as either 4 or 5, while the Scale-level CVI (S-CVI) was calculated as the average of the I-CVI values across all items.

For the I-CVI, was calculated as the proportion of experts rating the item as 4 or 5 out of the total number of experts and an I-CVI ≥ 0.78 indicates excellent content validity [27, 28].

$$\:\mathrm{I}-\mathrm{C}\mathrm{V}\mathrm{I}=\frac{\mathrm{N}\mathrm{u}\mathrm{m}\mathrm{b}\mathrm{e}\mathrm{r}\:\mathrm{o}\mathrm{f}\:\mathrm{e}\mathrm{x}\mathrm{p}\mathrm{e}\mathrm{r}\mathrm{t}\mathrm{s}\:\mathrm{r}\mathrm{a}\mathrm{t}\mathrm{i}\mathrm{n}\mathrm{g}\:4\:\mathrm{o}\mathrm{r}\:5}{\mathrm{T}\mathrm{o}\mathrm{t}\mathrm{a}\mathrm{l}\:\mathrm{n}\mathrm{u}\mathrm{m}\mathrm{b}\mathrm{e}\mathrm{r}\:\mathrm{o}\mathrm{f}\:\mathrm{e}\mathrm{x}\mathrm{p}\mathrm{e}\mathrm{r}\mathrm{t}\mathrm{s}\:}$$

For the S-CVI, two indices were computed: the average method (S-CVI/Ave) and the universal agreement method (S-CVI/UA). The S-CVI/Ave was calculated as the mean of all I-CVI values across items: $\:\mathrm{S}-\mathrm{C}\mathrm{V}\mathrm{I}/\mathrm{A}\mathrm{v}\mathrm{e}=\frac{\sum\:_{i=1}^{k}I-CVI}{\mathrm{k}}$, where (k) represents the total number of items. The S-CVI/UA was calculated as the proportion of items that achieved universal agreement among experts (i.e., all experts rated the item as 4 or 5):

$$\begin{aligned}&\mathrm{S}-\mathrm{C}\mathrm{V}\mathrm{I}/\mathrm{U}\mathrm{A}\cr &=\frac{\mathrm{N}\mathrm{u}\mathrm{m}\mathrm{b}\mathrm{e}\mathrm{r}\:\mathrm{o}\mathrm{f}\:\mathrm{i}\mathrm{t}\mathrm{e}\mathrm{m}\mathrm{s}\:\mathrm{w}\mathrm{i}\mathrm{t}\mathrm{h}\:\mathrm{u}\mathrm{n}\mathrm{i}\mathrm{v}\mathrm{e}\mathrm{r}\mathrm{s}\mathrm{a}\mathrm{l}\:\mathrm{a}\mathrm{g}\mathrm{r}\mathrm{e}\mathrm{e}\mathrm{m}\mathrm{e}\mathrm{n}\mathrm{t}}{\mathrm{k}}\end{aligned}$$

An S-CVI/Ave value ≥ 0.90 and/or an S-CVI/UA value ≥ 0.80 is generally considered indicative of excellent content validity, demonstrating strong expert consensus regarding the relevance and clarity of the translated items [17].

For reliability analysis, test–retest reliability was examined using the intraclass correlation coefficient (ICC_3,1_), based on a two-way mixed-effects model with absolute agreement. The interpretive thresholds were as follows: ICC < 0.50 = poor, 0.50–0.75 = moderate, 0.75–0.90 = good, and > 0.90 = excellent reliability [28]. Cronbach’s alpha (α) was calculated to assess the internal consistency reliability of the LEFS-V. A Cronbach’s alpha between 0.70 and 0.95 considered acceptable [29], whereas a value greater than 0.95 may indicate item redundancy. To enhance the stability of measurement, the standard error of measurement (SEM) for the LEFS-V was calculated as follows: SEM = s_p_ * $\:\sqrt{(1-ICC)}$ in which s_p_ = $\:\sqrt{0.5*\:\left({s}_{1}^{2}+\:{s}_{2}^{2}\right)}$ with s_1_ - the standard deviation of the initial assessment and s_2_ - the standard deviation of the reassessment [29]. The minimal detectable change at the 95% confidence level (MDC₉₅) for the LEFS-V, representing the smallest change in score that can be interpreted as a true change beyond measurement error, was calculated using the following formula: MDC_95_ =1,96*$\:\sqrt{2\:}$*SEM [29].

Additionally, the study examined the responsiveness of the LEFS-V to change by calculating the floor and ceiling effects of the scale. These effects were determined by calculating the percentage of participants who achieved the lowest possible score (floor effect) and the highest possible score (ceiling effect). Floor and ceiling effects for the LEFS-V were considered acceptable when no more than 15% of participants achieved either the minimum or maximum score on the scale [17].

For construct validity, data from the LEFS-V, KOOS-V, and NPRS obtained at the initial assessment were used to determine convergent and discriminant validity. Spearman’s correlation coefficients (r_s_) were calculated to examine the relationships between the LEFS-V, NPRS, and KOOS-V subscales. Correlation strengths were interpreted as strong (r_s_ ≥ 0.50), moderate (0.35 ≤ r_s_ < 0.50), and weak (r_s_ < 0.35) [30, 31].

## Results

### Translation and cross-cultural adaptation

Twenty items of the LEFS were translated into Vietnamese following the cross-cultural adaptation procedure proposed by Beaton et al. (1989). The five steps of cross-cultural adaptation (Fig. 1) were completed, and most items showed consistent translations between the two forward translators. However, several items, including A3, A10, and A12, required discussion to reach a unified translation consensus. The pre-final version of the LEFS-V was pilot tested on 10 individuals with KOA who met the inclusion and exclusion criteria described earlier. The results were very positive, as most participants understood and answered the questions easily without any apparent confusion. In a few cases, participants paused briefly to clarify certain items. For example, for item A7 (“Lifting an object, like a bag of groceries from the floor”), participants asked about the approximate weight. The researchers explained that it referred to a typical grocery bag containing a few small items such as vegetables, tomatoes, or apples. Similarly, for item A11 (“Walking 2 blocks”), participants asked about the distance, which was clarified as roughly equivalent to walking a short distance, such as from the beginning to the end of an alley. The remaining items did not require further clarification. Additionally, the average completion time of approximately five minutes indicated that the questionnaire was simple and quick to complete. After discussion and consensus with the expert committee, it was decided to retain the original wording of the pre-final version as the final LEFS-V for psychometric property assessment, since only two out of ten participants had expressed minor uncertainty about those two items.

### Content validity

The LEFS-V demonstrated very high content validity, with most items showing complete agreement among experts regarding their appropriateness and clarity. Although a few items did not achieve absolute unanimity, the agreement rate remained above 83%, with an S-CVI/Ave of 0.97 and an S-CVI/UA of 0.75. These findings indicate that the scale comprehensively captures lower limb functional performance within the Vietnamese cultural context. Details of the content validity analysis are presented in Table 1.

**Table 1:** Content validity index of LEFS-V

Items	Experts												No. of agreement	I - CVI	UA
	ID 01	ID 02	ID 03	ID 04	ID 05	ID 06	ID 07	ID 08	ID 09	ID 10	ID 11	ID 12			
A1	1	1	1	1	1	1	1	1	1	1	1	1	12/12	1	1
A2	1	1	0	1	1	1	1	1	1	1	1	1	11/12	0.92	0
A3	1	1	1	1	1	1	1	1	1	1	1	1	12/12	1	1
A4	1	1	1	1	1	1	1	1	1	1	1	1	12/12	1	1
A5	1	1	1	1	1	1	1	1	1	1	1	1	12/12	1	1
A6	1	1	1	1	1	1	1	1	1	1	1	1	12/12	1	1
A7	1	1	1	1	1	1	1	1	1	1	1	1	12/12	1	1
A8	1	1	0	1	1	0	1	1	1	1	1	1	10/12	0.83	0
A9	1	1	0	1	1	0	1	1	1	1	1	1	10/12	0.83	0
A10	1	1	1	1	1	1	1	1	1	1	1	1	12/12	1	1
A11	1	1	1	1	1	1	1	1	0	1	1	1	11/12	0.92	0
A12	1	1	1	1	1	1	1	1	1	1	1	1	12/12	1	1
A13	1	1	1	1	1	1	1	1	1	1	1	1	12/12	1	1
A14	1	1	1	1	1	1	1	1	1	1	1	1	12/12	1	1
A15	1	1	1	1	1	1	1	1	1	1	1	1	12/12	1	1
A16	1	1	1	1	1	1	1	1	1	1	1	1	12/12	1	1
A17	1	1	1	1	1	1	1	1	1	1	1	1	12/12	1	1
A18	1	1	1	1	1	1	1	1	1	1	1	1	12/12	1	1
A19	1	1	1	1	1	1	1	1	1	1	1	1	12/12	1	1
A20	1	1	1	1	1	1	1	1	0	1	1	1	11/12	0.92	0
**S-CVI/Ave**														**0.97**	
**S-CVI/UA**															**0.75**

Abbreviation: I-CVI; item-level content validity index, UA; universal agreement among experts, S-CVI; scale-level content validity index

### Test-retest reliability and internal consistency

Table 2 presents the demographic characteristics and assessment results of individuals with KOA. Of the 174 participants, 75 were included in the test–retest reliability analysis and completed the LEFS-V, NPRS, and KOOS-V during the initial assessment. Among them, 14 participants reported a change of more than two points on the NPRS, leaving 61 participants who completed the LEFS-V for the retest. The results demonstrated excellent reliability, with an ICC (3,1) of 0.985 (95% CI: 0.974–0.991) and a Cronbach’s alpha of 0.942. The SEM and MDC₉₅ values were 1.4 and 3.88 points, respectively, as shown in Table 3.

**Table 2:** Demographics and assessments of individuals with knee OA

Variables	*N* (%)	Mean ± SD
**Age (year)**	174 (100)	67.92 ± 9.67
**Gender**,** n (%)**		
Male	36 (20.7)	
Female	138 (79.3)	
**BMI**,** n (%)**		
Lean to Normal (< 25.0 kg/m^2^)	66 (38.0)	
Overweight (25.0–29.9 kg/m^2^)	50 (28.7)	
Obesity (≥ 30.0 kg/m^2^)	58 (33.3)	
**Education Levels**,** n (%)**		
Primary and secondary schools	52 (29.9)	
Highschool	100 (57.5)	
Graduated and above	22 (12.6)	
**Occupation**,** n (%)**		
Manual worker	53 (30.5)	
Office worker	7 (4.0)	
Retired	114 (65.5)	
**Comorbidity**,** n (%)**		
Yes	96 (55.2)	
No	78 (44.8)	
**Affected side**,** n (%)**		
Left	25 (14.4)	
Right	23 (13.2)	
Both sides	126 (72.4)	
**Duration of knee OA (year)**	174 (100)	5.01 ± 4.75
**LEFS-V**		
Initial assessment	174 (100)	39.94 ± 11.95
Reassessment	61 (35.1)	42.77 ± 11.03
**NPRS**		
Initial assessment	174 (100)	4.89 ± 1.34
Reassessment	61 (35.1)	4.79 ± 0.89
**KOOS**		
KOOS-Pain	174 (100)	65.91 ± 10.38
KOOS-Symptom	174 (100)	69.28 ± 9.33
KOOS-ADL	174 (100)	43.49 ± 17.99
KOOS-Sport/Rec	174 (100)	37.64 ± 17.23
KOOS-QoL	174 (100)	52.09 ± 9.98

Abbreviation: LEFS-V; Lower Extremity Functional Scale Vietnamese version, NPRS; Numerical Pain Rating Scale, KOOS; Knee injury and Osteoarthritis Outcomes Scale, ADL; activities of daily living, QoL; quality of life

**Table 3:** Test-retest reliability and Internal consistency of LEFS-V

Reliability	Cronbach’s alpha (α)	ICC _(3,1)_ (95%CI)	SEM	MDC_95_
Test-retest	0.985	0.985 (0.974–0.991)	1.4	3.88
Internal consistency	0.942	-	-	-

Abbreviation: ICC; intraclass correlation coefficient, SEM; standard error of measurement, MDC; minimal detectable change at the 95% confidence level

### Floor and ceiling effect

Among the 174 participants included in the study, no floor or ceiling effects were observed, as only 0.6% of participants (*n* = 1/174) achieved the maximum score and 0.6% (*n* = 1/174) achieved the minimum score on the LEFS-V. These findings indicate that the scale is suitable and sensitive for assessing lower limb function in the studied population, allowing data collection across the full range of functional variability.

### Construct validity

We used the Vietnamese version of the KOOS to compare with the LEFS-V, to assess the convergent and discriminant validity of the scale. The correlation coefficients between LEFS-V with NPRS and KOOS-V are shown in Table 4.

**Table 4:** Spearman’s correlation coefficient (r_s_) between the LEFS-V and NPRS, KOOS-V

Variable	NPRS	KOOS-Pain	KOOS-Symptom	KOOS-ADL	KOOS-Sport/Rec	KOOS-QoL
LEFS-V	-0.63**	0.59**	0.30**	0.71**	0.50**	0.18*

Abbreviation: LEFS-V; Lower Extremity Functional Scale Vietnamese version, NPRS; Numerical Pain Rating Scale, KOOS; Knee injury and Osteoarthritis Outcomes Scale, ADL; activities of daily living, QoL; quality of life

*p < 0.05; **p < 0.01

## Discussion

For the translation and cross-cultural adaptation, each step was conducted independently, including forward and backward translation, synthesis of the translations, and expert panel consensus to compare the first draft of the LEFS-V with the original version. This process was consistent with previous studies on LEFS translation into various languages, which followed Beaton’s standardized guidelines for cross-cultural adaptation [14]. However, several minor procedural differences were noted. In the Italian version, three initial forward translations were performed, and an interdisciplinary panel of nine experts reviewed the terminology [7]. In contrast, for the Dutch version, the original developer was directly involved in reviewing and approving all stages of the translation process and the final version [10].

### Cross-cultural adaptation and content validity

This study followed the five steps of Beaton’s guidelines for translation and cross-cultural adaptation, employing two independent forward translators with no prior experience using the instrument and two backward translators who were unfamiliar with the scale and not involved in its measurement domain. A panel of twelve interdisciplinary experts demonstrated a high level of consensus in evaluating the LEFS-V translation. The final Vietnamese version of the LEFS (LEFS-V) was developed through this standardized, multi-step translation process, similar to other language adaptations. The LEFS-V achieved semantic equivalence with the original version, ensuring linguistic clarity, cultural appropriateness, and conceptual consistency within the Vietnamese context.

In this study, the LEFS was collected through face-to-face interviews. Based on the results of the pilot study, most participants were older adults and preferred an interview format, which also helped ensure more accurate responses. A pilot study involving 10 individuals with knee OA was conducted prior to the main study to develop a standardized operational procedure (SOP) for the interview process, including agreement on how interviewers should ask the questions (i.e. weight of grocery and distance of walking) and how the items should be interpreted when communicating with participants.

Based on the original LEFS, most of the wording in each item was straightforward to translate into another language without significant linguistic barriers. However, a common challenge in translation lies in addressing phrases or activities that may not fully correspond to the target culture. During the LEFS-V translation process, similar issues were encountered in three items–A3, A10, and A12–which required careful cultural and linguistic consideration to ensure conceptual equivalence.

Firstly, regarding item A3, “Getting in and out of the bathtub” some experts have suggested changing the term “bathtub” to “bathroom” because bathtubs are not common in Vietnam. However, there are still some opposing opinions, arguing that bathtubs are not so rare that they need to be replaced. After discussion, the expert panel decided to retain the original translation to ensure maximum conceptual similarity with the original scale and to avoid altering the construct intended to be measured. Moreover, this translation still ensures the ability to cover functional activities in the bathroom, while maintaining consistency when comparing international research results. In contrast, in the Brazilian adaptation of the LEFS, item A3 was modified to *“Stepping over an obstacle approximately 50 cm high (e.g.*,* getting in/out of the bathtub)”* to reflect local relevance. The authors justified this change by noting that very few families in Brazil use bathtubs [12] highlighting the importance of local cultural adaptation during translation.

Secondly, for item A10, *“Getting into and out of a car*,*”* the translators agreed to make an addition. They proposed including the phrase *“Getting on and off a motorbike”* in the LEFS-V to ensure cultural relevance, as motorbikes are the most common means of transportation in Vietnam. In addition, the phrase *“Getting into and out of a car”* was retained. Importantly, the original item referring to “getting into and out of a car” was not replaced; the motorbike activity was added only to improve cultural relevance. During the interview, participants were asked to rate the difficulty based on the type of vehicle they most commonly use in their daily lives. The translators and experts agreed that this modification did not alter the conceptual meaning or structure of the original item; rather, it allowed respondents to better relate the question to real-life situations, thereby enhancing clarity and applicability. This adaptation may differ from other language versions, where motorbike use is not a common cultural practice.

Lastly, for item A12, *“Walking a mile*,*”* the translators agreed to clarify the unit of distance by adding its metric equivalent, resulting in *“Walking a mile (approximately 1.6 kilometers)”.* This adjustment helped Vietnamese respondents visualize the walking distance more easily. However, the translators decided not to replace “a mile” entirely with “1.6 kilometers” to preserve the original terminology and maintain conceptual equivalence while enhancing cultural acceptability. The unit of measurement in item A12 has also been modified in several other cross-cultural adaptations of the LEFS. For example, in the Spanish version, the word “*mile*” was replaced with “*kilometer*.” Similarly, in the Italian, Turkish, Persian, and Brazilian Portuguese versions, one mile was substituted with one kilometer to improve clarity and local relevance. In contrast, the Dutch version converted one mile to its approximate equivalent of 1.5 km to preserve the original intent of the English version [7–12, 14].

In addition, some less common local activities were reworded to better reflect culturally appropriate expressions. Despite these minor adjustments, no items were removed from the scale. All modifications were carefully made to preserve the original meaning of each measurement item while ensuring comprehensibility for local respondents and cultural relevance within the Vietnamese context. It is noteworthy that a common feature among previous LEFS translation studies is their consistent application to patient populations with similar characteristics in terms of age, type of knee or hip osteoarthritis, or other lower-limb musculoskeletal disorders.

### Psychometric properties of LEFS-V

In this study, questionnaires were administered through face-to-face interviews rather than self-administered forms. This approach has also been observed in several studies in the Brazilian and Italian versions [8, 12], some other versions do not explicitly mention how they approached the subjects through interviews or self-administration. The original LEFS approach does not prescribe a specific mode of administration, as previous studies have employed both interviewer-administered and self-administered formats depending on the research design and characteristics of the study population. Therefore, the psychometric properties observed in this study largely support the use of LEFS-V in face-to-face interview settings. Future studies could further investigate its psychometric properties when used as a self-administered questionnaire on different musculoskeletal populations.

For psychometric properties, numerous previous studies have demonstrated excellent reliability and validity across all translated versions of the LEFS (including the Vietnamese version of LEFS) and equivalent to the original English version.

The results demonstrated excellent internal consistency of the LEFS-V, with a Cronbach’s α of 0.942. This finding is consistent with other translated versions of the LEFS, such as the Spanish (Cronbach’s α = 0.99), Italian (Cronbach’s α = 0.94), and Dutch versions (Cronbach’s α = 0.94–0.96) [8, 10, 11]. Similarly, other adaptations have also reported high internal consistency values, including the Serbian (α = 0.95), Brazilian Portuguese (α = 0.95), Arabic (α = 0.95), and Simplified Chinese versions (α = 0.97) [7–10, 12, 32]. Thus, most translated versions of the LEFS have shown Cronbach’s α values ranging from 0.94 to 0.99, indicating that the items within the scale are highly interrelated and that the instrument consistently retains the same measurement structure as the original English version. However, this may indicate the possibility of redundance items when compared to previous translation and LEFS validation studies [10, 12, 33, 34]. It is also understandable that, among the 20 items of the LEFS, there is variation that covers a range of functional dimensions, which helps to avoid redundancy; for example, items A4, A11, and A12 all relate to walking ability but represent different levels or contexts of functional performance.

In addition, the test–retest reliability of the LEFS across different language versions has been consistently high, often reaching ICC values ≥ 0.86 and exceeding the accepted threshold of 0.70, despite variations in study populations [30]. For example, the Brazilian Portuguese version reported an ICC of 0.96 (retest after 2 days) among individuals with knee injuries, while the Spanish version demonstrated an ICC of 0.99 (retest after 2–5 days) in participants with lower-extremity dysfunction [10, 12]. Other translations have also shown similarly high ICC values: the Italian version (ICC = 0.89–0.91), Dutch version (ICC = 0.86), Arabic version (ICC = 0.96), and Chinese version (ICC = 0.97), with most studies focusing on populations with KOA or other musculoskeletal conditions [7, 8, 11, 32]. A similar pattern was observed in the present study, with an ICC of 0.94 (95% CI: 0.90–0.95) among individuals with KOA. Such high ICC values confirm that the LEFS demonstrates excellent stability and reliability across different patient populations and cultural contexts.

In our study, the MDC95 value of LEFS-V was 3.88, lower than the MDC90 value of 8 points reported in the original study [5]. This difference can be explained by the different confidence levels used and differences in sample characteristics and measurement conditions. Although we used a higher confidence level (95%), the MDC value in this study was still smaller than the lower confidence level (90%) of the original author, indicating lower measurement error and higher measurement accuracy in LEFS-V. This finding suggests that LEFS-V may be more sensitive in detecting smaller changes in functional status in individuals with knee osteoarthritis.

For construct validity, studies of the LEFS in multiple languages have demonstrated that the scale correlates strongly with other measures of physical function and shows strong negative correlations with pain intensity scales, thereby confirming both convergent and discriminant validity. In the Italian version, LEFS scores correlated strongly with the SF-36 Physical Functioning subscale (*r* = 0.77–0.78) at pre- and post-discharge, while showing only weak correlations with the SF-36 Mental Health subscale (*r* = 0.19–0.24), suggesting that the LEFS effectively measures lower-limb physical function and is clearly distinct from mental health constructs [8]. Similarly, the Dutch version reported high correlation coefficients with the WOMAC Function subscale (*r* = 0.81) and the KOOS-ADL subscale (*r* = 0.78), further supporting the convergent validity of the instrument [11].

In this study, the KOOS-V and NPRS scales were used to examine the construct validity of the LEFS-V. The results showed a strong positive correlation with the KOOS-ADL subscale, moderate correlations with the KOOS-Pain and KOOS-Sport/Rec subscales, a weak correlation with the KOOS-Symptom and KOOS-QoL, and a strong negative correlation with the NPRS. These findings are theoretically consistent because the LEFS-V primarily measures lower extremity functional performance in daily and sports-related activities, which conceptually overlap with the KOOS-ADL and KOOS-Sport/Rec domains. The moderate correlation with KOOS-Pain reflects the relationship between pain and functional limitations. In contrast, the weaker correlations with KOOS-Symptom and KOOS-QoL suggest that the LEFS-V focuses more on physical function than on symptom severity or overall quality of life. These findings confirm the convergent and discriminant validity of the LEFS-V, consistent with results reported in other language versions. Similarly, the Serbian version demonstrated very high correlations between the LEFS and the SF-36 Physical Functioning subscale (*r* = 0.89), as well as strong negative correlations with the Lequesne Index (*r* = − 0.98) and NPRS (*r* = − 0.76) [33]. The Persian version also reported higher correlations between the Persian LEFS and the SF-36 Physical Health component (*r* = 0.78) than with the SF-36 Mental Health component (*r* = 0.39), confirming that the LEFS is a sensitive instrument for assessing the degree of functional impairment in patients with KOA [14]. Overall, evidence from multiple countries and languages shows that the LEFS has strong convergent validity when compared with standardized functional scales (SF-36 PF, KOOS-ADL, WOMAC, IKDC) and strong discriminant validity when compared with pain scales (NRS, VAS, Lequesne). These results are consistent with the LEFS-V version, thereby confirming that the LEFS is a reliable and valid tool for measuring lower limb function in clinical practice and multicenter research.

This study has several limitations. First, it evaluated the LEFS-V at a single time point, which precluded the assessment of its responsiveness to clinical changes over time. Second, the study sample included only individuals with knee osteoarthritis; recruited from patients undergoing rehabilitation at the hospital’s Rehabilitation Department and at religious orders, who met the inclusion criteria and voluntarily agreed to participate in the study. Therefore, the generalizability of the findings to other musculoskeletal disorders of the lower extremities remains uncertain. Furthermore, the questionnaires were administered through standardized face-to-face interviews rather than self-administered forms. While this method helps ensure accurate understanding of the questionnaire items in older participants, our psychometric results largely support the use of LEFS-V in face-to-face interview settings. Finally, objective measurements such as the Timed Up and Go test (TUG) or the Sit-to-Stand test (STS) were not included to further substantiate the construct validity of the LEFS-V. Future research should adopt longitudinal designs to evaluate the responsiveness and minimal clinically important difference (MCID) of the LEFS-V, thereby supporting its application in monitoring functional recovery over time. Furthermore, integrating objective performance-based measures such as the TUG or STS would strengthen the evidence for construct validity by examining the relationship between self-reported and performance-based functional outcomes.

## Conclusion

The Vietnamese version of the LEFS (LEFS-V) meets all key criteria for a self-reported outcome measure, with appropriate linguistic and cultural adaptation for the Vietnamese context (see in Appendix 1). In this study, the scale was implemented through direct interviews with a standardized question structure to assist elderly patients or those with difficulty completing the questionnaire themselves. The scale demonstrates excellent internal consistency and test–retest reliability, as well as clear convergent and discriminant validity, thereby confirming its construct validity. These psychometric properties support the LEFS-V as a reliable and practical instrument for evaluating lower-limb function in both scientific research and clinical practice among Vietnamese-speaking populations with KOA.

## Appendix 1

**THANG ĐO CH**Ứ**C NĂNG CHI D**ƯỚ**I**.

Chúng tôi muốn tìm hiểu liệu bạn có gặp bất kỳ khó khăn nào khi thực hiện các hoạt động dưới đây do vấn đề ở chi dưới mà bạn hiện đang cần được hỗ trợ. Vui lòng cung cấp câu trả lời cho từng hoạt động.

Hôm nay, bạn có hoặc sẽ có bất kỳ khó khăn nào trong việc:

**Table:** 

	Hoạt động	Rất khó khăn hoặc không thể thực hiện.	Khá khăn nhiều.	Khó khăn vừa phải.	Khó khăn một chút.	Không khó khăn.
1	Bất kỳ công việc thường ngày, việc nhà, hoặc hoạt động ở trường nào của bạn.	0≤	1≤	2≤	3≤	4≤
2	Các sở thích, hoạt động giải trí hoặc thể thao mà bạn thường làm.	0≤	1≤	2≤	3≤	4≤
3	Việc vào hoặc ra khỏi bồn tắm.	0≤	1≤	2≤	3≤	4≤
4	Đi lại giữa các phòng.	0≤	1≤	2≤	3≤	4≤
5	Mang giày hoặc mang tất (vớ).	0≤	1≤	2≤	3≤	4≤
6	Ngồi xổm.	0≤	1≤	2≤	3≤	4≤
7	Nâng một vật, ví dụ như túi đồ từ sàn.	0≤	1≤	2≤	3≤	4≤
8	Thực hiện các công việc nhẹ nhàng trong nhà.	0≤	1≤	2≤	3≤	4≤
9	Thực hiện các công việc nặng nhọc trong nhà.	0≤	1≤	2≤	3≤	4≤
10	Ra vào ô tô hoặc lên xuống xe máy.	0≤	1≤	2≤	3≤	4≤
11	Đi bộ 2 dãy nhà.	0≤	1≤	2≤	3≤	4≤
12	Đi bộ 1 dặm (khoảng 1,6 km).	0≤	1≤	2≤	3≤	4≤
13	Lên hoặc xuống 10 bậc thang (khoảng 1 tầng lầu).	0≤	1≤	2≤	3≤	4≤
14	Đứng trong 1 giờ.	0≤	1≤	2≤	3≤	4≤
15	Ngồi trong 1 giờ.	0≤	1≤	2≤	3≤	4≤
16	Chạy trên mặt đất phẳng.	0≤	1≤	2≤	3≤	4≤
17	Chạy trên mặt đất gồ ghề.	0≤	1≤	2≤	3≤	4≤
18	Chuyển hướng đột ngột khi chạy nhanh.	0≤	1≤	2≤	3≤	4≤
19	Nhảy lò cò.	0≤	1≤	2≤	3≤	4≤
20	Lăn mình trên giường.	0≤	1≤	2≤	3≤	4≤
	**TỔNG ĐIỂM THEO CỘT**:					
**Mức thay đổi tối thiểu có thể phát hiện (Độ tin cậy 90%): 9 điểm**		**TỔNG ĐIỂM: __/80 (Điền tổng điểm từ các câu trả lời của bạn vào chỗ trống)**				

Source: Binkley et al. (1999): The Lower Extremity Functional Scale (LEFS): Scale development, measurement properties, and clinical application. Physical Therapy. 79:371–383

## Data Availability

The datasets generated and/or analyzed during the current study are not publicly available due to organizational confidential but are available from the corresponding author on reasonable request.

## Abbreviations

ADL: Activities of Daily Living
CI: Confidence Interval
I-CVI: Item-Content Validity Index
ICC: Intraclass Correlation Coefficience
KOA: Knee Osteoarthritis
KOOS: Knee Injury and Osteoarthritis Outcome Scale
LEFS: Lower Extremity Functional Scale
NICE: The National Institute for Health and Clinical Excellence
NPRS: Numerical Pain Rating Scale
MDC_95_: Minimum Detectable Change
PROMs: Patient-reported outcome measures
SEM: Standard Error of Measurement
SF-36: 36-Item Short Form Health Survey
STS: Sit to Stand
TUG: Time Up and Go
